# Factors Associated with Poor Quality of Life in Breast Cancer Survivors: A 3-Year Follow-Up Study

**DOI:** 10.3390/cancers15245809

**Published:** 2023-12-12

**Authors:** Soo-Hyun Kim, Ha-Yeon Jo

**Affiliations:** Department of Nursing, Inha University, Incheon 22212, Republic of Korea; dddus92@inha.edu

**Keywords:** breast cancer, depression, fatigue, insomnia, quality of life

## Abstract

**Simple Summary:**

Although quality of life (QOL) improves over time for most breast cancer survivors (BCSs), specific subgroup of BCSs may show persistently low or deteriorated QOL even after their treatment ends. Early identification of high-risk groups for QOL deterioration is crucial for quality cancer care. This study was conducted to identify subgroups of QOL change among 101 Korean BCSs, and to examine factors associated with subgroups that show poor QOL. During a 3-year observation, 22.6% of the participants showed consistently low or deteriorated QOL. Persistent symptoms of insomnia, fatigue, anxiety, depression, and comorbidity were found to be significant risk factors of poor QOL in BCS. These findings are helpful to identify high-risk groups for QOL deterioration among BCSs.

**Abstract:**

The purpose of this study was to identify subgroups of quality of life (QOL) changes in breast cancer survivors (BCSs), and to determine factors associated with subgroups of consistently low or deteriorated QOL. We enrolled 101 women recently diagnosed with breast cancer in South Korea and asked them to complete a questionnaire at baseline (within 1 month of diagnosis), 1 year later (Year 1), 2 years later (Year 2), and 3 years later (Year 3). We assessed QOL using the global QOL subscale from the EORTC QLQ-C30. We defined low QOL as a global QOL score 10 points below the mean score of the general population. Based on low QOL as defined in this study, we identified subgroups of QOL changes over 3 years. We identified four subgroups of QOL changes: improved (47.4%), stable (30%), continuously low (8.8%), and deteriorated (13.8%), and considered the last two categories (22.6%) poor QOL. Logistic regression analyses demonstrated that significant determinants of poor QOL were insomnia at Year 1, fatigue and anxiety at Year 2, and fatigue, depression, and comorbidity at Year 3. In conclusion, persistent symptoms of insomnia, fatigue, anxiety, depression, and comorbidity are potential risk factors for poor QOL in BCSs.

## 1. Introduction

Breast cancer is the most common female cancer in South Korea, as it is worldwide [1]. Due to advances in early detection and treatment, the relative 5-year survival rate for breast cancer survivors (BCSs) in high-income countries exceeds 90% [2], resulting in a constantly growing number of long-term survivors. As the number of BCSs has increased, quality of life (QOL) issues have become a vital consideration for care in this population.

Previous studies reveal that a substantial portion of BCSs suffer from long-term or late effects of cancer treatment, including pain, fatigue, insomnia, and lymphedema [3,4], ultimately decreasing their QOL [4]. In several large population-based studies, QOL in post-treatment BCSs was significantly lower than that in the general population [5,6,7]. Longitudinal studies on QOL among BCSs indicate that overall, QOL tends to deteriorate during the first year post diagnosis and improve in the long term [8,9,10,11,12,13,14].

The QOL of many BCSs recovers to that of the general population between 1 and 3 years post diagnosis [11,15]; however, specific groups continue to have a low or deteriorated QOL [16,17,18]. Di Meglio et al. [18] characterized long-term QOL trajectories in a large sample (*n* = 4131) of BCSs. During 4 years of follow-up, 10% of participants (*n* = 413) showed a deteriorated QOL, and 6.6% (*n* = 272) showed a consistently low QOL. Similarly, Goyal et al. [17] reported that 14.2% (93/653) of a BCS cohort showed a consistently low QOL 18 months after a breast cancer diagnosis. In a study of Korean BCSs (*n* = 126) [16], 58.9% had a consistently low QOL from pre-chemotherapy to 6 and 12 months after chemotherapy’s completion. Such subgroups that show consistently low or deteriorated QOL should be a prioritized target for survivorship care. Thus, identification of characteristics associated with this subgroup (hereafter defined as the “poor QOL group”) is crucial. 

Previous studies revealed that younger age, low economic status, excess body weight, comorbidity, endocrine therapy, current smoking, insufficient physical activity, and depression have been associated with long-term poor QOL [16,17,18]. Psychological factors, including low social support, passive coping, and less optimism have been associated [17]. Few studies, however, have examined the association of long-term BCSs’ symptoms with poor QOL. 

We have reported our longitudinal observations of coping style and QOL—and their association from diagnosis to 3 years post-diagnosis—among 101 BCSs [19]. Using those cohort data, we conducted a second analysis to better understand QOL changes and their correlates. The purpose of the current study was to identify subgroups of QOL changes in BCSs over 3 years after diagnosis, and to determine factors associated with this poor QOL group, including sociodemographic, clinical, lifestyle, and symptom variables.

## 2. Materials and Methods

### 2.1. Study Design and Participants

**Figure 1:**
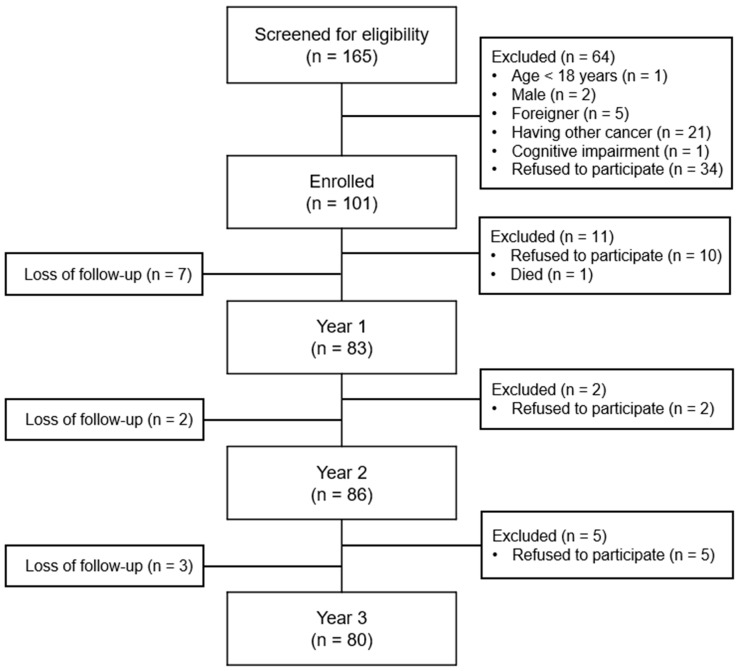
Flow of the study participants.

This is a secondary analysis of a longitudinal study [19] that examined the association between coping style at diagnosis and subsequent QOL for 3 years among BCSs. Included were women aged ≥19 years who had received a first-time diagnosis of breast cancer within 1 month, had a plan for cancer treatment, and were able to read and write Korean. Women were excluded if they had other cancer(s), had cognitive impairment, or were already undergoing cancer treatment. We screened 165 women and enrolled 101. The retention rate was 82.2% (*n* = 83) at 1 year after enrolment (‘Year 1’), 85.1% (*n* = 86) at 2 years after enrolment (‘Year 2’), and 79.2% (*n* = 80) at 3 years after enrolment (‘Year 3’). A detailed participant flow is presented in Figure 1.

### 2.2. Data Collection

Between 2010 and 2011, after approval from the Institutional Review Board of Inha University Hospital in South Korea, participants were recruited within 1 month of breast cancer diagnosis. Physicians screened subjects for their interest in participating in this study, which a research nurse explained in face-to-face interviews. Women who agreed to participate were provided with an informed consent form followed by a questionnaire. Data collection was conducted 1, 2, and 3 years after enrolment. Follow-up questionnaires were mailed to participants to complete and return to the research team. Clinical information was obtained through electronic medical records after completion of primary treatment. Participants were followed until 2015.

### 2.3. Measures

We measured physical symptoms and financial impact using the symptom subscales and items from the European Organization for Research and Treatment of Cancer Quality of Life Questionnaire-C30 (EORTC QLQ-C30), including fatigue (3 items), nausea/vomiting (2 items), pain (2 items), dyspnea (1 item), insomnia (1 item), appetite loss (1 item), constipation (1 item), diarrhea (1 item), and financial difficulties (1 item). The scoring principle and imputation of missing values follow the EORTC scoring manual [20]. Scores ranges from 0 to 100, with higher scores representing more severe symptoms. The Korean version of the EORTC QLQ-C30 has been validated [21]. In this study, the Cronbach’s alpha of the three subscales ranged from 0.62 to 0.72. In order to analyze symptom variables as binary variables, we dichotomized them using the thresholds for clinical importance (TCIs) developed by Giesinger et al. [22]. The TCIs for the symptom variables were fatigue (39), nausea/vomiting (8), pain (25), dyspnea (17), insomnia (50), appetite loss (50), constipation (50), diarrhea (17), and financial difficulties (17). These TCIs were used in a previous BCS study [18].

We measured QOL using the global QOL subscale (2 items) from the Korean version of EORTC QLQ-C30 [21]. The scoring principle and imputation of missing values follow the EORTC scoring manual [20]. The score ranges from 0 to 100, with a higher score representing a better QOL. In this study, the Cronbach alpha of the global QOL subscale was 0.85. To analyze the QOL as a binary variable, we dichotomized it using normative reference data. We based our binary QOL variable on whether the global QOL score had fallen more than the minimal clinically important difference (MCID) below the Korean female general population mean of 67.7 [23]. The MCID of the EORTC QLQ-C30 used was 10 points, in keeping with previous studies [24,25] and resulting in a score of 57.7 for the study cut-off. Thus, a low QOL was defined as a global QOL score of <57.7.

We measured psychological symptoms using the Korean version of the Hospital Anxiety and Depression Scale (HADS) [26]. The HADS consists of two subscales with anxiety (7 items) and depression (7 items), each on a 4-point Likert scale (0–3). The score is obtained by summing the scores within each subscale, which ranged from 0 to 21. A higher score denotes more severe symptoms. In this study, the Cronbach’s alpha was 0.86 for the anxiety subscale and 0.80 for the depression subscale. In order to analyze symptom variables as binary variables, we used a cut-off score of ≥8 for each subscale, as in a previously validated study [27].

Sociodemographic variables that we collected included age, marital status, educational level, religion, employment status, monthly income, menopausal status, and comorbidity. Clinical variables included cancer stage at diagnosis, type of surgery, and type of treatments (chemotherapy, radiation therapy, and anti-hormone therapy). Lifestyle variables included regular exercise (yes/no), tobacco use behavior (current, former, never smoking), and alcohol consumption behavior (yes/no). 

### 2.4. Statistical Analysis

In this longitudinal study, we analysed data from at least three time points (i.e., baseline, Year 1 and/or Year 2, and Year 3), resulting in a dataset of 80 participants. We analyzed data with SPSS 26.0 software (SPSS Inc., Chicago, IL, USA), using standard descriptive measures.

We identified 4 subgroups of QOL changes. For the “improved” group, baseline QOL scores were <57.7 but increased to ≥57.7 at Year 3, regardless of Year 1 or Year 2 scores. For the “stable” group, baseline QOL scores were ≥57.7 and maintained until Year 3. For the “continuously low” group, baseline QOL scores were <57.7 and maintained until Year 3. For the “deteriorated” group, baseline QOL scores were ≥57.7 but decreased to <57.7 at Year 3, regardless of Year 1 or Year 2 scores. In this study, we clustered the “consistently low” and “deteriorated” QOL groups as the “poor” QOL group.

We performed univariate logistic regression analyses at three time points (Year 1, Year 2, and Year 3), calculating odds ratio (OR) and corresponding 95% confidence intervals (CIs) to identify potential correlates of poor QOL. Variables with a *p*-value < 0.05 in the univariate analyses at each time point were included in the corresponding multivariable logistic regression model. For assessing multicollinearity, we analysed the correlation coefficients among potential predictors with magnitudes of 0.80 or higher. We obtained best-fitting multivariable logistic regression models using a forward stepwise elimination procedure. We reported the adjusted OR and 95% CIs for the final models. The significance level was set at *p* < 0.05, and all significance tests were two-sided.

## 3. Results

### 3.1. Characteristics of the Participants

Table 1 presents the Year 3 sociodemographic and clinical characteristics of the participants. The mean age of the participants was 54.1 years (SD = 8.9), with most (43.7%) in their 50 s. All women received surgery; 73.8% underwent breast-conserving surgery. The majority received chemotherapy (73.8%) or radiation therapy (71.3%).

### 3.2. Lifestyle and Symptoms Characteristics over Time

Table 2 displays lifestyle and symptom characteristics for 3 years after diagnosis. More than 60% maintained regular exercise across three time points, and the number who smoked decreased remarkably. Alcohol consumption behavior, on the other hand, worsened over time, with the number of drinkers increasing.

EORTC QLQ-C30 symptom results showed that fatigue, nausea/vomiting, pain, dyspnea, and insomnia were the most common symptoms across the three time points (ranging from 17.5% to 35.0%). The prevalence of these symptoms did not decrease; even the prevalence of fatigue, nausea/vomiting, and pain at Year 3 was the same or slightly greater than at Year 1. Financial difficulties were highly reported at all three time points but decreased gradually over time. The prevalence of anxiety ranged from 23.8% to 32.5%, and that of depression ranged from 23.8% to 28.7%. Two psychological morbidities improved slightly at Year 3 compared with Year 1 (Table 2).

### 3.3. QOL Change Subgroups

Table 3 shows the frequency of low-QOL cases over time and their patterns. At baseline, 56.4% had a low QOL, but the frequency gradually decreased to 33.7% at Year 1, 23.3% at Year 2, and 22.5% at Year 3. Among the QOL change subgroups, 47.4% showed an improved QOL, 30% were stable, 8.8% showed a continuously low QOL, and the QOL of 13.8% deteriorated. Thus, 22.6% of cases showed a poor QOL.

### 3.4. Univariate Logistic Regression Analyses for Poor QOL

Among sociodemographic and clinical factors, only comorbidity was significantly associated with poor QOL. Type of surgery showed borderline significance (*p* = 0.052), while no lifestyle factors showed any association. Among symptom variables, dyspnea, insomnia, and anxiety at Year 1; fatigue, pain, insomnia, anxiety, and depression at Year 2; and all symptoms except for diarrhea at Year 3 were significantly associated with poor QOL (Table 4). Due to their low frequency, smoking and constipation were not available for analysis at Year 3.

### 3.5. Multivariable Logistic Regression Analyses for Poor QOL

Multivariable logistic regression analyses for poor QOL were conducted at three time points. Before conducting the analyses, multicollinearity was checked using a correlation matrix between potential predictors. No multicollinearity problem was found. The correlation coefficients between potential predictors for the Year 1 model ranged from −0.223 to 0.329, those for the Year 2 model ranged from −0.270 to 0.511, and those for the Year 3 model ranged from 0.225 to 0.498.

In the model for Year 1, only insomnia was significantly associated with poor QOL; in the model for Year 2, fatigue and anxiety were significantly associated with poor QOL; and in the model for Year 3, comorbidity, fatigue, and depression were significantly associated with poor QOL (Table 5).

## 4. Discussion

Although QOL improves over time for most BCSs, some women may have consistently low or deteriorated QOL (defined as poor QOL in this study). We identified subgroups of QOL changes using longitudinal cohort data and sought to examine characteristics that relate to the poor QOL group. We believe that our results provide important evidence for the development of a care program for those survivors who are at risk of a poor QOL during their follow-up period.

The four subgroups of QOL changes that we identified among BCSs were characterized by improved (47.4%), stable (30.0%), consistently low (8.8%), and deteriorated (13.8%) QOL. Interestingly, in Di Meglio et al.’s study [18] of 4131 BCSs, the patterns of QOL changes were quite similar to ours, although their sample was larger: excellent (51.7%), very good (31.7%), deteriorated (10.0%), and continuously low (6.6%). Goyal et al. [17] identified six subgroups of QOL changes among 653 BCSs: consistently high (36.0%), recovery to high (10.3%), consistently medium (26.5%), recovery to medium (13.0%), consistently low (10.3%), and consistently very low (4.0%). Considering our results along with previous studies, approximately 15–20% of BCSs may be at risk of poor QOL after cancer treatment. These women constitute a prioritized target for a survivorship care programs.

On the other hand, we found a marked difference in the proportion of survivors in the consistently low group (8.8% vs. 58.9%) compared with Park et al.’s study of 126 BCSs [16]. This discrepancy might result from the difference in treatment characteristics of the participants; all Park et al.’s [16] subjects had received chemotherapy [16], while ours had not. In addition, they tracked QOL after chemotherapy and at 6 and 12 months after its completion, while we followed-up QOL at 1, 2, and 3 years after diagnosis. This means that Park et al.’s [16] sample would have been more significantly affected by treatment than ours, which might have caused the higher proportion of those individuals in the consistently low QOL group.

Early identification of high-risk groups for poor QOL is crucial for prevention of QOL deterioration. From this clinical perspective, our findings provide important results by exploring potential risk factors for poor QOL at each time point. Among Year 1 variables, we found insomnia to significantly increase risk of poor QOL, which was consistent with Lee et al. [8], who also found that increased insomnia among BCS was significantly associated with deteriorated QOL 1 year after diagnosis. Insomnia is one of the five most burdensome long-term issues in BCS [28]. In the current study, the prevalence of insomnia was highest at Year 1 (22.5%), and persisted. Insomnia is a major side effect of anti-hormone therapy (e.g., tamoxifen). Many BCSs initiate this therapy during the first year, and this might cause insomnia. Natural menopause may also be a cause. In South Korea, the most common age for breast cancer diagnosis is 40–50 years, so menopause might be the cause of insomnia in many. Our results suggest that screening for insomnia and appropriate interventions should be required, particularly in the first year of treatment. Evidence-based guidelines from the National Comprehensive Cancer Network (NCCN) [29] recommend cognitive behavioral treatments (e.g., relaxation therapy, stimulus control, sleep restriction) for insomnia in cancer survivors. Such approaches are preferred over pharmacologic interventions as a first choice [29].

Among Year 2 variables, we found fatigue and anxiety to significantly increase poor QOL risk. Fatigue is one of the most distressing symptoms among all cancer survivors, and approximately a third of BCSs report chronic fatigue [30]. In this study, the prevalence of fatigue was highest at Year 2 (28.7%) and decreased to 22.5% at Year 3, which was slightly higher than at Year 1 (21.3%). Our results support the finding that fatigue persists even after cancer treatment ends. Consistent with our findings, Lee et al. [8] reported that persistent fatigue among BCSs was a significant predictor of deteriorated QOL. Currently, fatigue management is well established, and therapies, including physical activity, psychoeducational intervention, mindfulness-based stress reduction (MBSR), and cognitive behavioral therapy [29], need to be applied more actively in clinical practice.

Many cancer survivors do not have psychiatric clinical diagnoses, but still have psychological symptoms such as anxiety and depression, which can have a negative impact on QOL. Anxiety or depression affects up to 29% of cancer survivors, and the symptoms do not necessarily resolve with time [29]. At Year 1 in our study, 32.5% of BCSs had anxiety, and 28.7% had depression. This prevalence did not resolve at Year 3. Anxiety was found to be a risk factor for poor QOL at Year 2, as was depression at Year 3. Those findings were in line with previous longitudinal studies [10,16,17]. In a 14-month longitudinal study on QOL among 83 BCSs, Hsiao et al. [10] found that increased anxiety and depression predicted worse QOL. Appropriate interventions include a strong recommendation of regular physical activity [29]. In fact, evidence suggests that exercise may be as effective as antidepressants in the treatment of depression [31]. Other alternative treatments include yoga, tai chi, and MBSR [29]. Mindfulness is possibly the best-studied alternative treatment for psychological distress in cancer survivors. For example, in a randomized controlled trial among 322 BCSs, Lengacher et al. [32] found that a 6-week MBSR program significantly reduced anxiety and fear of recurrence.

Among Year 3 variables, comorbidity, fatigue, and depression significantly increased risk of poor QOL. Comorbidity is an important issue among cancer survivors because cancer and/or its treatment increases the risk of comorbidities such as cardiovascular disease, diabetes mellitus, and osteoporosis [33]. Several cross-sectional studies of BCSs have revealed comorbidity severity to be associated with a decrease in QOL [9,34,35]. In Schoormans et al.’s study of BCSs [35], the negative impact of comorbidity on QOL increased with time after diagnosis. Specifically, cardiovascular disease and depression were the strongest associates. Among longitudinal studies, Di Meglio et al. [18] also found severity of comorbidity to be significantly associated with deteriorated QOL. Longitudinal studies about the impact on QOL of specific types of comorbidities, however, are still lacking. Further research is required.

Unexpectedly, we could not find anything significant regarding lifestyle factors. Di Meglio et al. [18] found that insufficient physical activity and tobacco smoking were significantly associated with deteriorated QOL, suggesting the importance of a healthy lifestyle. The small sample size in our study may have diminished its power to detect significant results. Moreover, the number of smoking women was too small for analysis. Due to these methodological flaws, definite conclusions cannot be drawn.

Our finding of no significant sociodemographic-, disease- or treatment-related factors was unexpected. Prior longitudinal studies demonstrated that sociodemographic factors such as age [14,16,18,34], education [8,17], economic status [16,18], and employment status [8] were significant correlates of continuously low or deteriorated QOL. In addition, clinical factors such as chemotherapy [8,17], type of surgery [10], and endocrine therapy [18] were found to have a significant correlation with QOL. From a clinical perspective, those findings yield a better understanding of where particular attention could be given to specific groups, such as survivors who are younger and/or belong to a low social class. This study’s non-significant findings may be attributed to the small sample size. Hence, further research with larger sample sizes is needed.

### 4.1. Implications

Our finding of the poor QOL of post-treatment BCSs is evident from symptom problems. Since persistent insomnia, fatigue, anxiety, and depression were found to increase the risk of poor QOL, their periodic screening and effective interventions should be incorporated into clinical practice. Since comorbidity may also increase poor QOL risk, referrals should be made during the survivorship period. Our results may be valuable for guiding the development of a survivorship care program for post-treatment BCSs.

### 4.2. Limitations

This study had several limitations. First, the study sample was enrolled from a single institution in South Korea, which limits its generalizability. Second, our sample size was relatively small, so it may have not detected low-frequency subgroups of QOL changes, thus increasing the likelihood of a Type II error. Future studies from other institutions and larger sample sizes are required. Third, given its secondary analysis nature, this study did not include other psychological factors (e.g., coping, social support, self-efficacy) likely to affect QOL change. Fourth, the EORTC QLQ-C30 used in this study is not a breast cancer-specific measurement, which may limit our ability to identify the QOL of breast cancer survivors. Lastly, our data are somewhat outdated. Thus, caution is needed in the interpretation of the study results.

## 5. Conclusions

This study identified subgroups of QOL changes in BCSs for 3 years after diagnosis. Most BCSs maintained or recovered their QOL over time, but 22.6% showed continuously low or deteriorated QOL. Persistent symptoms, such as insomnia, fatigue, anxiety, and depression, as well as comorbidity, were found to be potential risk factors for continuously low or deteriorated QOL. The findings offer clinically important insight for the development of interventions to preserve QOL in post-treatment BCSs, including periodic symptom assessment and management, as well as timely referral for comorbidity management.

## Figures and Tables

**Table 1:** General characteristics of the study participants (*n* = 80).

Characteristics	Category	*n* (%)
Age (years)	<40	3 (3.8)
	40–49	22 (27.5)
	50–59	35 (43.7)
	≥60	20 (25.0)
Marital status	Married	65 (81.3)
	Single	3 (3.7)
	Divorced/widowed	12 (15.0)
Education (*n* = 79)	Elementary school	9 (11.3)
	Middle school	17 (21.3)
	High school	42 (52.5)
	College or university	11 (13.8)
Religion	No	32 (40.0)
	Yes	48 (60.0)
Employment status	No	46 (58.2)
	Yes	33 (41.8)
Monthly income (*n* = 78)	<$2000	28 (35.9)
	≥$2000	50 (64.1)
Menopause (*n* = 77)	No	12 (15.6)
	Yes	65 (84.4)
Comorbidity ^1^	No	50 (62.5)
	Yes	30 (37.5)
	Hypertension	13 (16.3)
	Diabetes	3 (3.8)
	Cardiovascular	4 (5.0)
	Musculoskeletal	5 (6.3)
	Thyroid disease	2 (2.5)
	Liver disease	2 (2.5)
	Pulmonary disease	1 (1.3)
	Others	4 (5.0)
Cancer stage at diagnosis	0	7 (8.7)
	1	29 (36.3)
	2	36 (45.0)
	3	8 (10.0)
Type of surgery	Mastectomy	21 (26.3)
	Breast-conserving surgery	59 (73.8)
Chemotherapy	No	22 (27.5)
	Yes	58 (72.5)
Radiation therapy	No	23 (28.7)
	Yes	57 (71.3)
Anti-hormone therapy	No	18 (22.5)
	Yes	62 (77.5)

^1^ Multiple responses were possible.

**Table 2:** Lifestyle and symptoms characteristics over time (*n* = 80).

Variables	Year 1	Year 2	Year 3
	*n* (%)		
Lifestyle			
Regular exercise	50 (62.5)	51 (63.7)	51 (63.7)
Tobacco use	8 (10.0)	3 (3.8)	1 (1.3)
Alcohol consumption	11 (13.8)	13 (16.3)	16 (20.0)
Symptoms			
EORTC QLQ-C30			
Fatigue	17 (21.3)	23 (28.7)	18 (22.5)
Nausea/vomiting	20 (25.0)	16 (20.0)	20 (25.0)
Pain	26 (32.5)	24 (30.0)	26 (32.5)
Dyspnea	26 (32.5)	28 (35.0)	24 (30.0)
Insomnia	18 (22.5)	16 (20.0)	14 (17.5)
Appetite loss	3 (3.8)	5 (6.3)	4 (5.0)
Constipation	5 (6.3)	8 (10.0)	2 (2.5)
Diarrhea	16 (20.0)	15 (18.8)	16 (20.0)
Financial difficulties	40 (50.0)	38 (47.5)	33 (41.3)
HADS			
Anxiety	26 (32.5)	19 (23.8)	22 (27.5)
Depression	23 (28.7)	22 (27.5)	19 (23.8)

**Table 3:** Quality of life (QOL) change patterns.

	*n* (%)
Cases with low QOL	
Baseline (*n* = 101)	57 (56.4)
Year 1 (*n* = 83)	28 (33.7)
Year 2 (*n* = 86)	20 (23.3)
Year 3 (*n* = 80)	18 (22.6)
QOL change subgroups	
Improved	38 (47.4)
Stable	24 (30.0)
Continuously low	7 (8.8)
Deteriorated	11 (13.8)

**Table 4:** Univariate logistic regression analyses for poor QOL.

Variables		OR	95% CI	*p*
Sociodemographic				
Age ≥ 50 years		1.263	0.418–3.815	0.679
No spouse		0.968	0.272–3.446	0.960
Education < high school		1.584	0.524–4.790	0.415
No religion		1.062	0.362–3.111	0.913
Not employed		1.152	0.399–3.327	0.794
Monthly income < $2000		2.065	0.713–5.986	0.182
Menopausal		3.896	0.467–32.374	0.209
Having comorbidity		4.889	1.590–15.028	0.006
Clinical				
Stage 2–3 (ref. stage 0–1)		1.875	0.625–5.629	0.262
MRM (ref. BCS)		3.015	0.991–9.175	0.052
Receiving chemotherapy		2.209	0.572–8.540	0.251
Receiving radiation therapy		0.547	0.181–1.651	0.284
Receiving anti-hormone therapy		1.021	0.289–3.601	0.974
Lifestyle				
Year 1	Exercises regularly	1.015	0.037–3.356	0.980
	Smokes	1.167	0.214–6.348	0.858
	Drinks	0.313	0.037–2.653	0.287
Year 2	Exercises regularly	1.128	0.372–3.425	0.831
	Smokes	7.600	0.645–89.574	0.107
	Drinks	0.557	0.111–2.782	0.476
Year 3	Exercises regularly	0.871	0.280–2.709	0.811
	Drinks	0.677	0.169–2.705	0.581
Symptoms				
Year 1	Fatigue	1.603	0.479–5.367	0.444
	Nausea/vomiting	2.399	0.777–7.409	0.128
	Pain	2.647	0.900–7.790	0.077
	Dyspnea	3.594	1.208–10.688	0.021
	Insomnia	5.889	1.840–18.845	0.003
	Appetite loss	1.765	0.151–20.658	0.651
	Diarrhea	1.783	0.526–6.040	0.353
	Financial difficulties	1.000	0.350–2.586	>0.999
	Anxiety	3.594	1.208–10.688	0.021
	Depression	2.507	0.837–7.504	0.100
Year 2	Fatigue	13.520	3.938–46.423	<0.001
	Nausea/vomiting	2.600	0.790–8.555	0.116
	Pain	5.923	1.918–18.295	0.002
	Dyspnea	2.263	0.776–6.599	0.135
	Insomnia	3.747	1.149–12.221	0.028
	Appetite loss	6.000	0.919–39.185	0.061
	Diarrhea	2.000	0.582–6.867	0.271
	Financial difficulties	2.037	0.697–5.953	0.193
	Anxiety	7.361	2.290–23.664	0.001
	Depression	7.286	2.307–23.010	0.001
Year 3	Fatigue	8.281	2.518–27.231	0.001
	Nausea/vomiting	4.636	1.497–14.361	0.008
	Pain	4.924	1.620–14.996	0.005
	Dyspnea	5.923	1.918–18.275	0.002
	Insomnia	7.467	2.130–26.172	0.002
	Appetite loss	12.200	1.184–125.717	0.036
	Diarrhea	1.190	0.332–4.271	0.789
	Financial difficulties	2.120	0.732–6.136	0.166
	Anxiety	3.769	1.245–11.414	0.019
	Depression	7.361	2.290–23.664	0.001

QOL, quality of life, CI = confidence interval, OR = odds ratio, MRM = modified radical mastectomy, BCS = breast-conserving surgery.

**Table 5:** Multivariable logistic regression analyses for poor QOL.

Time Points	Variable	aOR	95% CI	*p*
Year 1	Insomnia	5.889	1.840–18.845	0.003
Year 2	Fatigue	22.783	4.493–115.515	<0.001
	Anxiety	13.985	2.627–74.461	0.002
Year 3	Comorbidity	5.056	1.275–20.051	0.021
	Fatigue	9.070	2.167–37.954	0.003
	Depression	6.540	1.641–26.069	0.008

QOL = quality of life, aOR = adjusted odds ratio, CI = confidence interval. Variables entered into the Year 1 model were type of surgery, dyspnea, insomnia, and anxiety; variables entered into the Year 2 model were type of surgery, fatigue, pain, insomnia, anxiety, and depression; and variables entered into the Year 3 model were type of surgery, comorbidity, fatigue, nausea/vomiting, pain, dyspnea, insomnia, appetite loss, anxiety, and depression.

## Data Availability

The data that support the findings of this study are available on request from the corresponding author. The data are not publicly available due to privacy and ethical constraints.
